# Post radiation chylous ascites: a case report

**DOI:** 10.1186/1757-1626-2-9393

**Published:** 2009-12-23

**Authors:** Vishal G Shelat, Garvi J Pandya, Asim Shabbir, Ravishankar K Diddapur

**Affiliations:** 1 Department of Surgery, Tan Tock Seng Hospital, 308433 Singapore; 2 Department of Medicine, National University Hospital, Singapore; 3 Department of Surgery, National University Hospital, Singapore

## Abstract

We report a 64 years old gentleman with unresectable right-sided retroperitoneal liposarcoma, who underwent radiotherapy & subsequently developed chylous ascites. He failed conservative management of chylous ascites and this was successfully managed with a peritoneovenous shunt. The pathophysiology and management of post radiational chylous ascites is discussed.

## Introduction

Chylous ascites is uncommon; but the incidence has been increasing. The common causes of chylous ascites are malignancy and cirrhosis. Radiotherapy is an infrequent cause of chylous ascites. The management of chylous ascites comprises treatment of the underlying condition and peritoneo-venous shunt surgery is reserved for highly selected cases where the conservative measures fail.

## Case presentation

**Figure 1:**
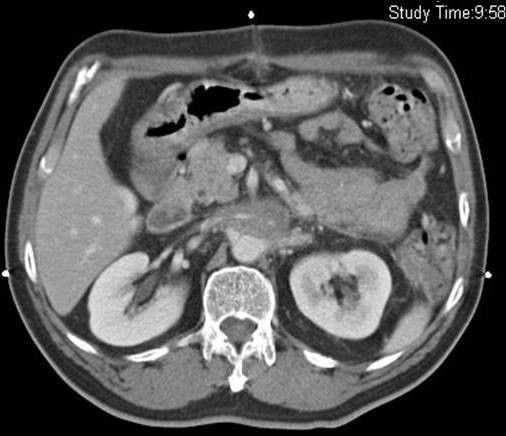
**Preoperative abdominal CT scan showing retroperitoneal mass encasing the right renal vein (arrow)**.

**Figure 2:**
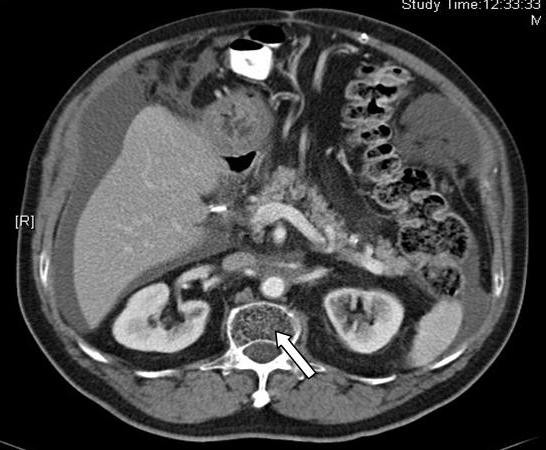
**Post radiotherapy CT scan showing reduction in tumour (arrow) with ascites**.

A 64-year-old Singaporean Chinese male was admitted to the hospital with non-specific abdominal pain. Physical examination was unremarkable. Computerized tomography (CT) scan of the abdomen showed a right sided retroperitoneal mass encasing the right renal vein (Figure. 1). He underwent an exploratory laparotomy. The lesion was found to be inoperable due to encasement of aorta and renal vessels and a biopsy was taken. Histology was consistent with a liposarcoma. He underwent radiotherapy to the lesion. The lesion responded well to radiotherapy and subsequently decreased in size. However, radiotherapy was complicated by the development of chylous ascites (Figure. 2). The presence of chylous ascites was confirmed by biochemical analysis after abdominal paracentesis. His ascites failed to resolve with conservative measures requiring fortnightly paracentesis. After six months of repeated paracentesis, in view of intractable and recurrent ascites, decision was made to offer a peritoneovenous shunt. A Denver shunt was performed and his ascites resolved. He is doing well at 6 months of follow-up without clinical or radiological evidence of ascites or shunt related complications.

## Discussion

Chylous ascites is the term used to describe milky ascitic chyle with high fat (triglycerides) contents, usually higher than 200 mg/dl. Morton first described it in an 18-months old child in 1619 [1] It is a rare condition with an incidence of 1 in 50,000 to 1 in 100,000 hospital admissions [2] The incidence has increased, probably due to better survival of cancer patients and more radical surgeries being performed commonly.

Multiple etiological factors can give rise to this condition including primary lymphatic diseases, malignancies, liver cirrhosis, infection (most commonly tuberculosis), retroperitoneal lymph node dissection, post-radiotherapy of retroperitoneum, pancreatitis and retroperitoneal fibrosis. In adults however, chylous ascites is associated most frequently with malignant conditions and more so from metastatic and disseminated carcinomas. Colonic, pancreatic, ovarian, testicular, renal and prostatic carcinomas are the common primary sites associated with the formation of chylous ascites due to tumour infiltrating and blocking the lymphatic vessels. Radiotherapy causes fibrosis of lymphatic vessels and ultimately obstruction. The obstruction of the lymphatic flow from the gut to cysterna chili causes a high pressure within the lymphatic vessels with subsequent subserosal leakage and chylous ascites [3].

Routine laboratory tests may show anemia, lymphocytopenia, hypoalbuminemia, raised liver enzymes and hyponatremia with a normal lipid panel. Confirmation of diagnosis is achieved by analysis of the ascitic fluid, which has a milky color with specific gravity raging from 1.010 to 1.054, elevated triglycerides, low cholesterol, high leukocytes count 232 -2560 cells/mm^3 ^and varying levels of protein contents [4]. In order to ascertain cause the use of investigations like, CT scan, barium studies, lymphangiogram, lymph node biopsy, bone marrow examination, intravenous pyelography and even exploratory laparotomy have been described [5,6].

Treatment of chylous ascites is directed at the underlying disease process along with symptomatic relief. Conservative management with high protein and low-fat diet with medium chain triglycerides may be successfully tried in selected cases. Medium chain triglycerides are absorbed directly into the intestinal cells and transported as free fatty acids & glycerol directly into the portal vein [5]. This bypasses the chylomicron to lymphatic transport as occurring with long chain triglycerides. Cirrhotic patients with chylous ascites are at high risk of developing encephalopathy with the above regime and hence salt & fluid restriction along with diuretics is preferable as initial management. When the above conservative measures fail, total parenteral nutrition may help by reducing the intestinal lymph flow. However, as in our case, where the lymphatic vessels are occluded, the chance of ascites resolving with conservative means is low and surgery may have to be considered in selected cases. Surgery to remove the obstructing lesion, ligating leaking lymphatics or resection of small bowel segments may either not be possible or may not yield good results [7]. Lymphangiography or lymphoscintigraphy may help identify the anatomical site of leak and provide a roadmap for the surgeon. Lymphatic microsurgery may be possible in selected cases where expertise is available. For patients with post radiation chylous ascites when life expectancy is limited because of underlying pathology a viable option would be regular abdominal paracentesis or large volume paracentesis (LVP). This however is associated with complications like, infection, malnutrition and lymphopenia. For patient who has intractable and recurrent ascites with a slow growing tumor and has failed conservative treatment, peritoneovenous shunting is a viable option. This is less invasive as compared to laparotomy for ligation of lymphatics. It also obviates the needs for repeated paracentesis and its associated complications. It benefits the patient by needing fewer visits to hospital with better life style and lesser biological/biochemical abnormalities. There are reports on postoperative chylous ascites being managed by peritoneovenous shunting [8]. The limitations of this technique are usually seen as shunt occlusion, disseminated intravascular coagulation, subclavian vein/superior vena cava thrombosis, shunt fracture, infection and may preclude a liver transplantation in suitable cases. Also, LVP may be as effective as a peritoneovenous shunt with no difference in survival [9].

Chylous ascites is a rare form of ascites, but incidence is increasing. Once diagnosed, paracentensis is indicated for symptomatic relief. Refractory and recurrent postradiational chylous ascites can be managed by peritoneovenous shunting with a good quality of life. Peritoneovenous shunting should be considered in well-selected cases when conservative measures fail.

## Consent

Written informed consent was obtained from the patient for publication of this case report and accompanying images. A copy of the written consent is available for review from the journal's Editor-in-Chief.

## Competing interests

The authors declare that they have no competing interests.

## Authors' contributions

GJP - Literature review and manuscript preparation, VGS - Manuscript preparation and Oversight, AS - medical management of patient and idea of publication with proposal to write and do early review of literature, RKD - Consultant in charge of the patient and oversight of the manuscript. All authors read and approved the final manuscript
